# Cytogenetic mechanisms of unisexuality in rock lizards

**DOI:** 10.1038/s41598-020-65686-7

**Published:** 2020-05-26

**Authors:** Victor Spangenberg, Marine Arakelyan, Marcelo de Bello Cioffi, Thomas Liehr, Ahmed Al-Rikabi, Elena Martynova, Felix Danielyan, Ilona Stepanyan, Eduard Galoyan, Oxana Kolomiets

**Affiliations:** 1 Vavilov Institute of General Genetics RAS, Moscow, Russia; 2 Department of Zoology, Yerevan State University, Yerevan, Armenia; 3 Laboratório de Citogenética de Peixes, Departamento de Genética e Evolução, Universidade Federal de São Carlos, São Carlos, SP Brazil; 4 Institute of Human Genetics, Jena University Hospital, Friedrich Schiller University, Jena, Germany; 5 Skolkovo Institute of Science and Technology, Moscow, Russia; 6 Scientific Center of Zoology and Hydroecology, Yerevan, Armenia; 7 Severtsov Institute of Ecology and Evolution RAS, Moscow, Russia

**Keywords:** Cytogenetics, Herpetology

## Abstract

*Darevskia* rock lizards is a unique complex taxa, including more than thirty species, seven of which are parthenogenetic. In mixed populations of *Darevskia* lizards, tri- and tetraploid forms can be found. The most important issues in the theory of reticulate evolution of *Darevskia* lizards are the origin of parthenogenetic species and their taxonomic position. However, there is little data on how meiosis proceeds in these species. The present work reports the complex results of cytogenetics in a diploid parthenogenetic species – *D. unisexualis*. Here we detail the meiotic prophase I progression and the specific features оf mitotic chromosomes organization. The stages of meiosis prophase I were investigated by immunocytochemical analysis of preparations obtained from isolated primary oocytes of *D. unisexualis* in comparison with maternal species *D. raddei nairensis*. It has been shown that in *D. unisexualis* at the leptotene-zygotene stages the axial elements and the synaptonemal complex (SC) form typical “bouquets”. At the pachytene-diplotene stage, 18 autosomal SC-bivalents and thickened asynapted sex Z and w univalents were observed. The presence of SYCP1 protein between the lateral elements of autosomal chromosomes proved the formation of assembled SCs. Comparative genomic hybridization (CGH) on the mitotic metaphase chromosomes of *D. unisexualis* was carried out using the genomic DNA isolated from the parental species *D. raddei nairensis* and *D. valentini*. In the pericentromeric regions of half of the mitotic chromosomes of *D. unisexualis*, specific regions inherited from maternal species have been found. Following our results, we suggest a model for diploid germ cells formation from diploid oocytes without premeiotic duplication of chromosomes in the oogenesis of diploid parthenogenetic lizards *D. unisexualis*. Taken as a whole, our findings confirm the hybrid nature of *D. unisexualis* and shed light on heterozygosity and automixis in diploid parthenogenetic forms.

## Introduction

Parthenogenetic reproduction in vertebrates was first discovered and later described by Ilya Darevsky in Caucasian rock lizards in 1957^1^. Later it was described in various snake and lizard groups^2^ and even showed that it can be induced in birds^3^. Most of the obligate vertebrate parthenogenetic forms have hybrid origin^4^. The hybrid origin of parthenogenetic species of rock lizards was established based on the data obtained in the skin transplantation experiments^5,6^, allozyme data^7–11^, as well as mitochondrial^12–15^ and nuclear DNA sequencing^16–21^. Due to their hybrid origin, parthenogenetic forms may differ in ploidy, with many species being triploid. However, parthenogenetic rock lizards (*Darevskia* spp.) are diploid^22^.

**Figure 1:**
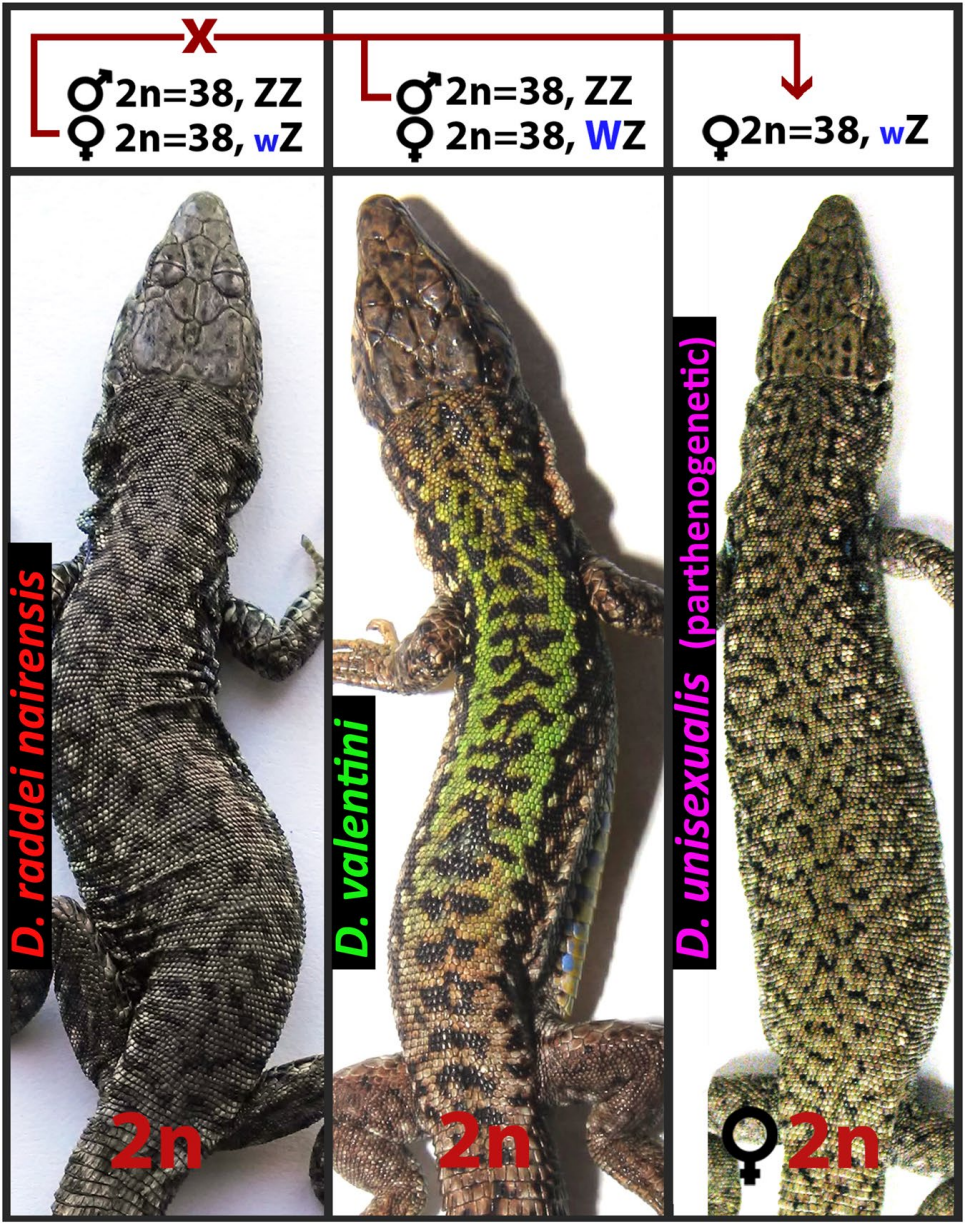
Scheme of the hybrid origin of diploid parthenogenetic species *D. unisexualis* (2n = 38) from the maternal species *D. raddei nairensis* and the paternal species *D. valentini*.

This work is focused on the parthenogenetic species *D. unisexualis* (Fig. 1). The hybrid origin of *D. unisexualis* from an ancestral cross between the maternal species *D. raddei nairensis* and paternal species *D. valentini* was revealed previously^7,8,12^.

In the Caucasus, there can be found seven diploid parthenogenetic species of rock lizards included within the *Darevskia* complex which shares similar karyotypes 2n = 38, with only one pair of microchromosomes^4^. But, notwithstanding the high value of knowledge on cytogenetic mechanisms underlying diploid parthenogenesis in lizards, it should be noted that only a small number of works are currently available addressing such issues.

Endoreplication preceding first meiotic division (premeiotic doubling) has been demonstrated for the whiptail lizard species with parthenogenesis belonging to the genus *Aspidoscelis* (formerly *Cnemidophorus*)^22–26^. Premeiotic endomitosis prevents irregular synapsis of homologous chromosomes at the first meiotic division and thus disruption of oogenesis is a consequence of the extremely high heterozygosity of hybrids. The involvement of the mechanisms of terminal fusion was shown for the snake species in the genus *Thamnophis* wherein only males are produced in the case of facultative parthenogenesis^27,28^. Facultative parthenogenesis was also described for two Varanidae species^29,30^, and the snake species in Acrochordidae, Crotalidae, Colubridae, and Pythonidae^27,31,32^, in which the oocyte nucleus and the second polar body fuse to reestablish diploidy.

In *Darevskia* rock lizards, Darevsky and Kulikova (1961, 1962, 1964)^33–35^ traced meiosis up to the metaphase of the second division and based on their findings put forward two hypotheses on how the restoration of diploid chromosome set may occur in parthenogenetic species. One of them stated the fusion of the second polar body with the oocyte nucleus during the anaphase of the second division and the other suggested fusion of the nuclei of the first cleavage division.

However, in a later work, Darevsky *et al*.^36^ noted that high heterozygosity levels observed in parthenogenetic rock lizards may challenge the hypothesis of suppression of the second meiotic division and support the hypothesis suggesting the fusion of pronucleus and the haploid descendant of the first polar body^36^. One more argument was suggested in favor of this hypothesis: unique submetacentric autosomal chromosome in the karyotype of the parthenogenetic species *D. rostombekowi* demonstrates no polymorphism in the populations of this species^36^. This is possible in the case of the reunification of the segregated chromosomes after the first meiotic division.

The endoduplication hypothesis was not excluded in the earlier studies^37^, however, this hypothesis was not able to explain the haploid number of bivalents previously detected during meiosis in parthenogenetic rock lizards^38^.

Synaptonemal complex (SC) karyotypes of *D. valentini* and *D. raddei* males comprise 19 acrocentric bivalents. Minor differences in chromosome pairing during the zygotene stage and certain differences in the average number of crossing-over sites have been described for these species^39^. Later, a species-specific SC-karyotype feature was found in one of the parental species – *D. raddei*, namely, the formation of additional centromere proteins signals localized near the native centromeres. Then, 3–9 dicentric chromosomes were found in the *D. raddei* spermatocytes I pachytene nuclei and the hypothesis of the epigenetic-based mechanism of additional neocentromeres formation has been proposed as a result^40^.

Sex chromosome identification in lacertids is highly important due to active evolution processes described for the ZW system in this group^41,42^ and the reptiles in general. Most works describe mitotic metaphase chromosomes^41–46^. Z and W lampbrush chromosomes were described for *Eremias velox*^47^. Studies on the sex chromosomes’ homology in the wide phylogenetic spectrum of lacertids and their outgroups revealed long-term evolutional stability of the ZW sex chromosome system across the lacertids^48^.

Previous cytogenetic studies of somatic karyotypes of four parental species of genus *Darevskia* revealed interspecific differences in the W chromosomes structure. Particularly, in two paternal species *D. valentini* and *D. portschinskii*, W chromosomes are heterochromatin-enriched macrochromosomes. At the same time, w chromosomes of the two maternal species *D. raddei nairensis* and *D. mixta* are shorter in length but contain large heterochromatic pericentromeric regions^49^. It was suggested by the authors that deletion in the euchromatic region of the W macrochromosome could lead to the formation of the w microchromosome in the maternal species, assuming that W macrochromosome is the ancestral, and w chromosome is the progressive type of sex chromosome. According to Kupriyanova (1997), *D. unisexualis* inherited the reduced w microchromosome from the maternal species *D. raddei nairensis*^50^.

The current work aimed to characterize the somatic karyotype (mitotic metaphase) as well as chromosome behavior during meiosis in the parthenogenetic rock lizard species *D. unisexualis*. Comparative genomic hybridization (CGH) on mitotic metaphase chromosomes of the parthenogenetic species *D. unisexualis* was performed. The study of meiotic cells was carried out to reveal the number of bivalents during all substages of prophase I, to study the specific features of homeologous chromosomes synapsis, as well as to identify Z and W sex chromosomes and investigate their behavior in meiosis. Towards this end, we performed the following procedures:

- Fluorescent labelling of the whole genomic DNA of *D. raddei nairensis* and *D. valentini* parental species and comparative genomic hybridization (CGH) on *D. unisexualis* mitotic metaphase chromosomes.
- Immunocytochemical study of the spread and squashed nuclei preparations of parthenogenetic *D. unisexualis* primary oocytes at the meiotic prophase I stage.

## Results

### Comparative genomic hybridization (CGH) on the metaphase plates of the parthenogenetic species *D. unisexualis* using the genomic fluorescence *in situ* hybridization (FISH) DNA probes corresponding to the parental species *D. raddei nairensis* and *D. valentini*

**Figure 2:**
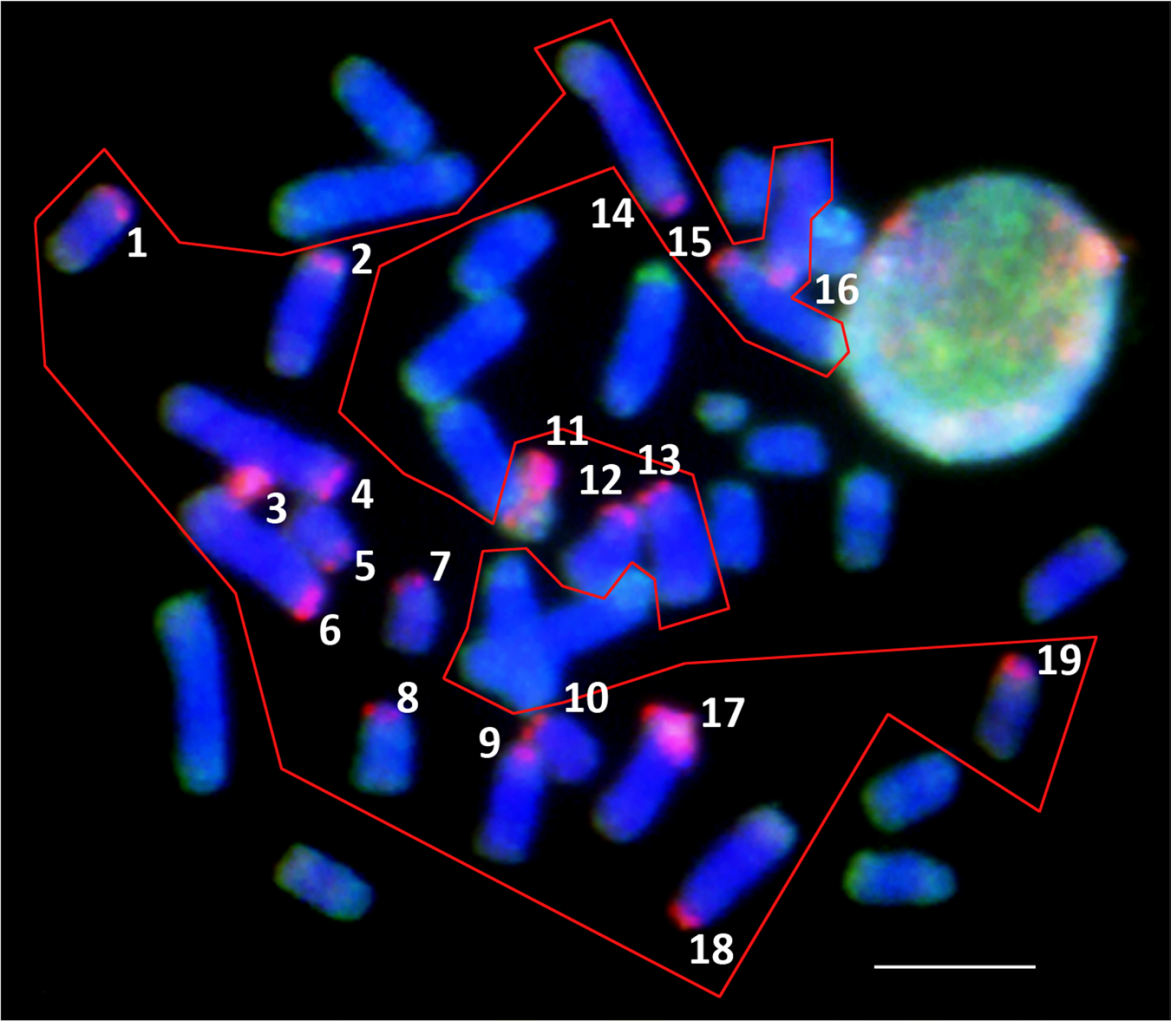
Comparative genomic hybridization (CGH) on the metaphase plate of the parthenogenetic species *D. unisexualis* (2 N = 38) with the DNA-FISH probes for the genomic DNA of the two parental species *D. raddei nairensis* (red) and *D. valentini* (green). Numbers 1–19 and the red line indicate chromosomes with the centromeric chromatin regions enriched with *D. raddei nairensis* gDNA. Bar = 5 μm.

One-half of the chromosomes (19 of the 38) of the parthenogenetic species *D. unisexualis* with diploid karyotype contains relatively large pericentromeric DNA fragments specific for the parental species *D. raddei nairensis* (chromatin fragments are shown in red) (Fig. 2). Despite the fact they share some repetitive DNA content in the same chromosome, the exclusive presence of highly accumulated repeated DNA (red and green signals) on 19 distinct chromosomes provides additional cytogenetic confirmation of the hybrid origin of the parthenogenetic species under study.

### Immunocytochemical study of the spread and squashed nuclei of bisexual species *D. raddei nairensis* primary oocytes

**Figure 3:**
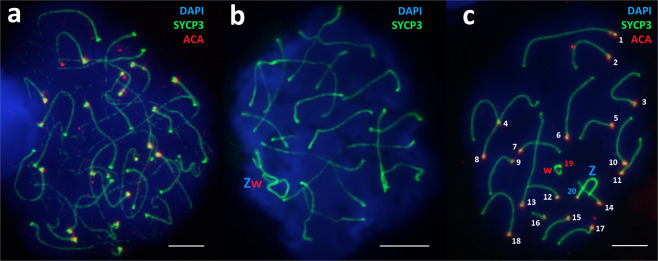
Spread nuclei of the bisexual species *D. raddei nairensis* primary oocytes at different stages of the meiotic prophase I. A – synaptonemal complexes assembly at zygotene; (**b,c**) – at pachytene stages. Sex Z and w chromosomes located closely (**b**) and asynaptic w and Z univalents (**c**). Bar = 5 μm.

We traced the assembly of synaptonemal complexes in the female of the maternal species *D. raddei nairensis* from zygotene (Fig. 3a) to pachytene (Fig. 3b,c). 18 autosomal bivalents and Z and w sex chromosomes were determined at the pachytene stage (Fig. 3c). Heteromorphic sex chromosomes have thickened axial elements and smaller centromere foci. We detected both variants of sex chromosomes location in the spread preparation of synaptonemal complexes: sex chromosomes located closely (Fig. 3b), and asynaptic w and Z univalents (Fig. 3c).

### Immunocytochemical study of the spread and squashed nuclei of diploid parthenogenetic species *D. unisexualis* primary oocytes

**Figure 4:**
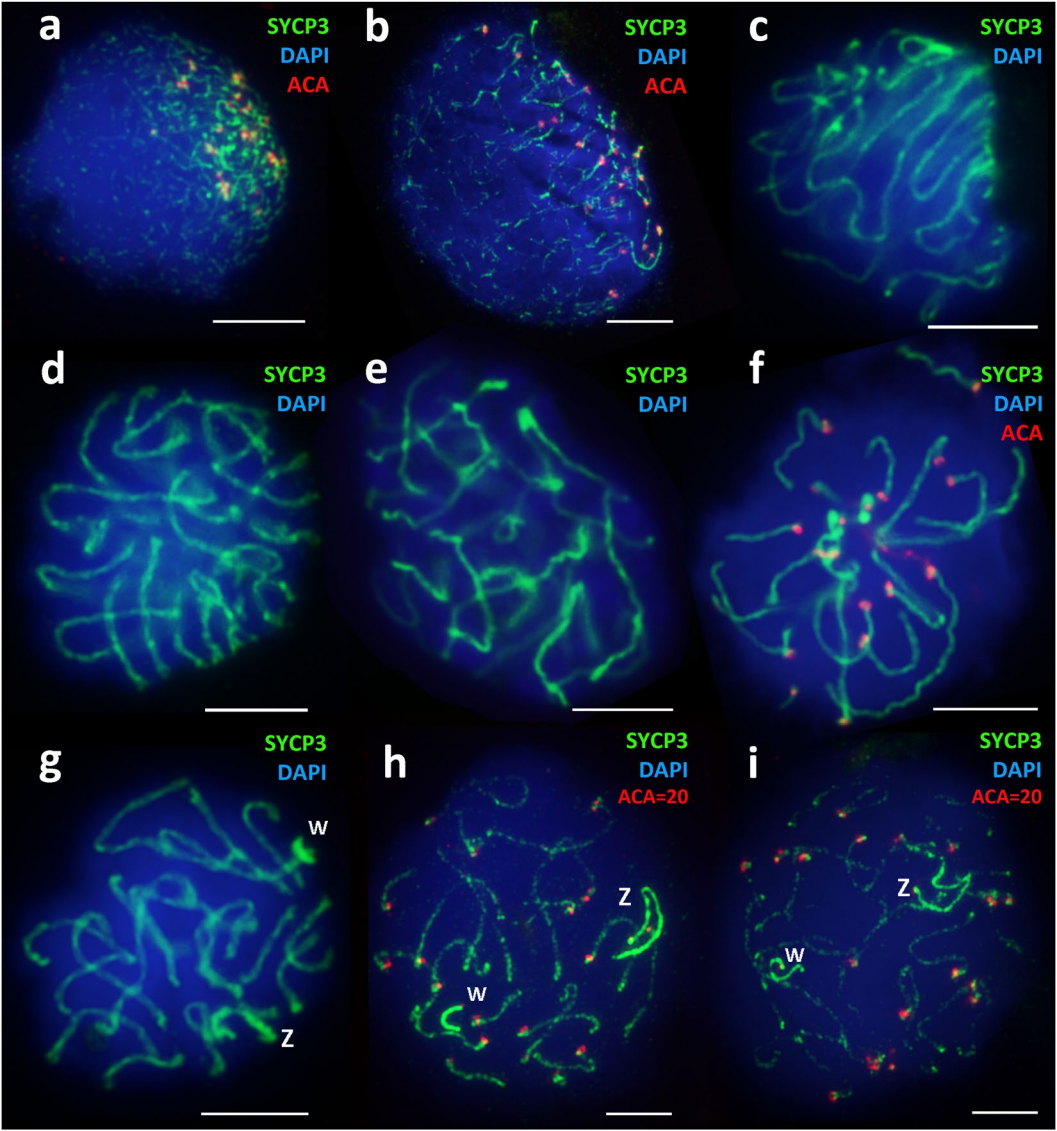
Spreaded nuclei of the diploid parthenogenetic species *D. unisexualis* primary oocytes at different stages of the meiotic prophase I. Synaptonemal complexes were stained with the antibodies against the SYCP3 protein (green), centromeres were stained with ACA antibodies (red), and chromatin was stained with DAPI (blue). (**a**) – late leptotene; (**b**,**c**) – early zygotene, the start of chromosome «bouquet» formation; (**d**) – late zygotene; (**e–f**) – pachytene; (**g**) – early diplotene, Z and w sex chromosomes – thickened axial elements; (**h,i**) – mid-diplotene. Degradation of the autosomal chromosomes synaptonemal complex axial structures starts before their desynapsis. Desynaptic zones are at the bivalents ends near to the pairs of ACA foci. Heteromorphic thickened axial elements of Z and w chromosomes are completely asynapted and lie at a considerable distance from each other. Bar = 5 μm.

Here, for the first time, we were able to obtain the preparations of oocyte nuclei from the parthenogenetic species *D. unisexualis*, squashed or spread at different stages of meiotic prophase I: from leptotene to mid-diplotene. Immunocytochemical studies of 103 primary oocyte nuclei were performed (Fig. 4).

Formation of the axial elements of meiotic chromosomes started at the late leptotene stage near one of the cell poles and was detected in 19 out of 103 analyzed nuclei (Fig. 4a). At the early zygotene stage, the assembly of the chromosome axial elements continues, and the formation of the “chromosome bouquet” structure begins (Fig. 4b).

**Figure 5:**
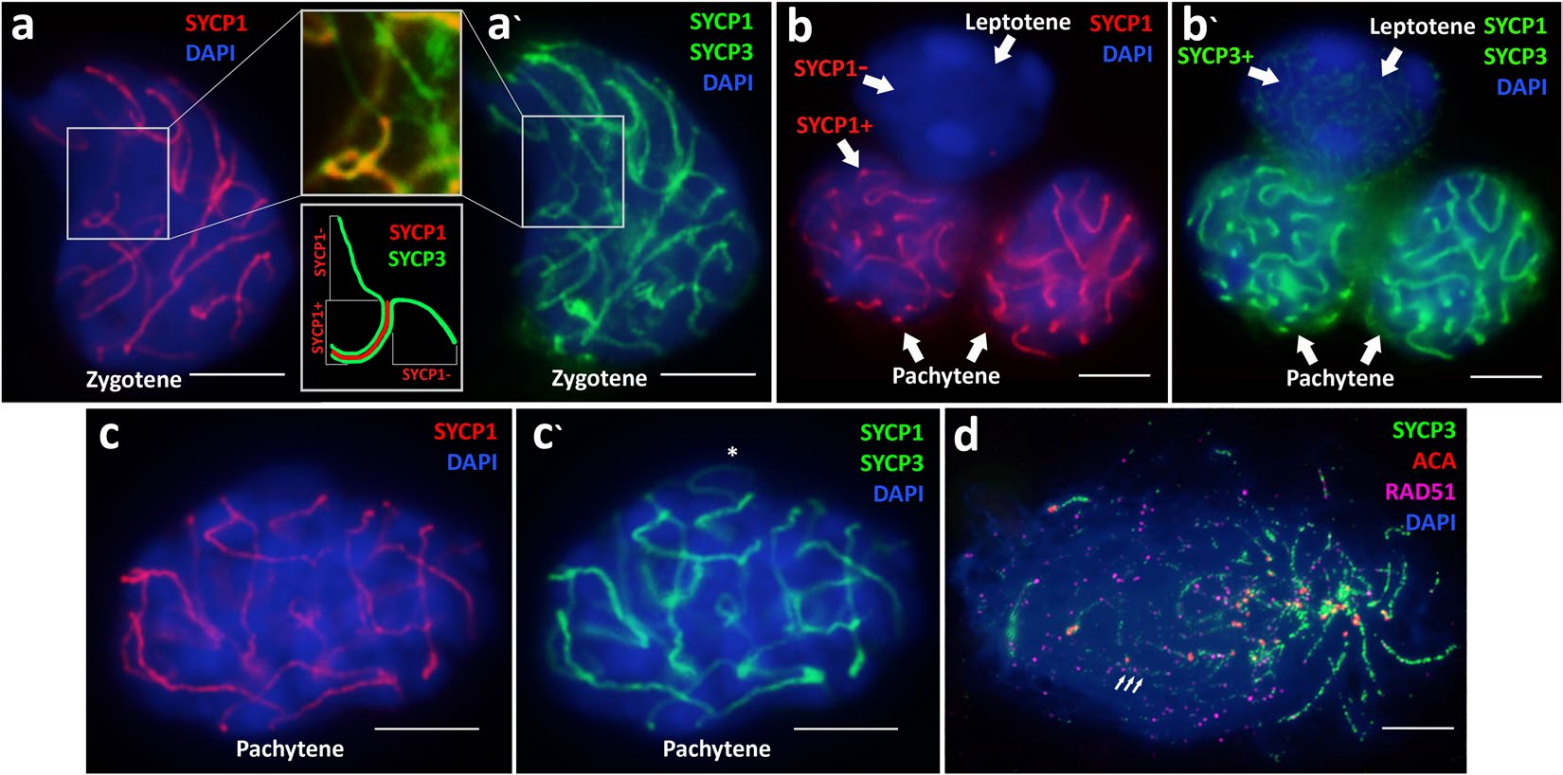
Synaptonemal complexes assembly and double-strand breaks (DSB) repair in *D. unisexualis*. Synaptonemal complexes assembly traced by sequential immunostaining with: (**a**–**c**) – anti-SYCP1 protein (central elements of SCs); and (**a‘**–**c‘**) – with the anti-SYCP3 protein antibodies (protein of SC axial elements) on the same nuclei from leptotene to pachytene. (**d**) – DNA DSB repair loci immunostained in the zygotene nucleus with the antibodies against the RAD51 protein (magenta). Chromatin was stained with DAPI (blue). Bar = 5 μm.

The “chromosome bouquet” (U-shaped chromosomes) is associated with the initiation of chromosome synapsis and is the hallmark of presynaptic stages of prophase I (Fig. 4a–c)^51,52^. At the mid-zygotene stage in *D. unisexualis*, the synapsis of the axial elements is elongated while maintaining the configuration of the “chromosome bouquet” (Fig. 4c–e). DNA double-strand breaks (DSB) repair loci were detected associated with the assembling axial elements of chromosomes at the early zygotene stage (Fig. 5d). Twenty-two primary oocyte nuclei at the early and mid-zygotene stages were studied.

To examine the synapsis of homeologous (inherited from the two parental species *D. raddei nairensis* and *D. valentini*) chromosomes and the assembly of synaptonemal complexes, we performed a detailed immunocytochemical study of *D. unisexualis* leptotene, zygotene, and pachytene stages (Fig. 5). For the immunocytochemical study, we used two antibodies combination which reactivity in reptiles has been experimentally proved by us in this study (see control antibodies test in the Supplementary Materials). Immunostaining with the anti-SYCP1 protein antibodies revealed the central element of the assembled synaptonemal complexes in all late zygotene (Fig. 5a) and pachytene (Fig. 5b) nuclei studied, but not in the nuclei at the leptotene stage (Fig. 5b). Subsequent immunostaining of the same nuclei with the anti-SYCP3 antibodies revealed asynapsed axial elements of the chromosomes at the leptotene stage (Fig. 5b‘) and partial asynaptic zones at the zygotene stage (Fig. 5a‘).

**Figure 6:**
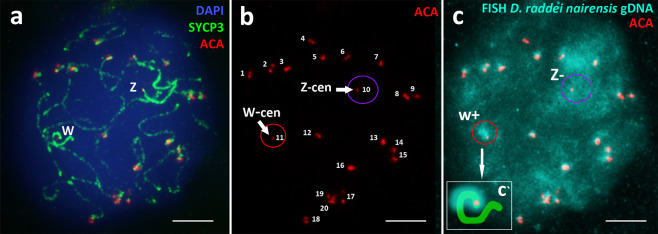
Sex chromosomes Z and w in the oocyte I spread preparation of *D. unisexualis*. Synaptonemal complexes were immunostained with antibodies against the SYCP3 protein (green) (**a**) and centromeres were immunostained with anti-kinetochore antibodies ACA (red) (**a,b**). Chromatin was stained with DAPI (blue). Asynaptic sex chromosomes have thickened axes, centromeres signals (w-cen and Z-cen) are different (small and not paired) from autosomal bivalents. FISH with maternal species *D. raddei nairensis* gDNA (**c**) revealed strong positive (W+) signal in the w chromosome pericentromeric region (scheme c‘) and negative (Z–) in Z chromosome. Bar = 5 μm.

The pachytene stage in *D. unisexualis* (17 nuclei studied) is characterized by the complete synapsis of autosomal chromosomes (Fig. 4e,f). The number of bivalents, as well as merged centromere signals, did not exceed 20 in the pachytene nuclei, which corresponds to the centromere signals on 18 autosomal bivalents and one smaller sized signal on each of Z and w chromosomes, respectively. The diplotene stage (45 nuclei studied) begins with the formation of short desynapsed zones at the ends of SC-bivalents (Fig. 4g). Intensive staining of thickened asynapted axial elements of Z and w sex chromosomes was obtained with the anti-SYCP3 protein antibodies in early diplotene (Fig. 4g). Then, there was a gradual fragmentation of the axial structures associated with SYCP3 protein unloading from the axial elements (Fig. 4h,i), which was also a diplotene stage-specific event in the males of both parental species^39^. At the diplotene stage, 18 desynapting autosomal bivalents and two completely asynaptic heteromorphic Z and w chromosomes are clearly visible, lying at a considerable distance from each other (Fig. 4h,i). These two chromosomes are characterized by a much stronger signal in the case of SYCP3 protein immunostaining (Figs. 4h,i and 6a) and by smaller centromere foci (Fig. 6b). FISH with the *D. raddei nairensis* gDNA probe revealed a strong specific signal in the pericentromeric region of the short w chromosome, but not of the Z chromosome (Fig. 6c). All the facts supporting the identity of these two chromosomes as Z and w sex chromosomes are listed in the DISCUSSION section below.

Therefore, the number of centromeres signals at the diplotene stage is 20, taking into account the initiation of desynapsis at the bivalent ends of some autosomes (Fig. 4g–i). Thus, the number of autosomal SC-bivalents detected in the parthenogenetic *D. unisexualis* is 18, which is typical for the meiotic prophase I of the parental bisexual rock lizard species. But unlike in males (ZZ), the heteromorphic asynapted axial elements of the Z and w chromosomes are clearly visible. None of the nuclei we studied at the diplotene stage showed signs of an increase in the number of chromosomes. This indicates the absence of the pre-meiotic chromosome doubling stage.

## Discussion

### Comparative genomic hybridization (CGH) on mitotic chromosomes of parthenogenetic *D. unisexualis*

The CGH method has been successfully implemented for the identification of parental genomes in hybrids/allopolyploids^53–58^. In the present work, the CGH results proved to be informative and allowed us to precisely characterize the hybridization process that led to the emergence of the parthenogenetic *D. unisexualis* species.

As a result of the CGH experiments performed on the parthenogenetic *D. unisexualis* mitotic metaphase chromosomes, we have found that one-half of the chromosomes (19 out of 38) of this species carry relatively large fragments of pericentromeric chromatin which is specific to the *D. raddei nairensis* parental species (Fig. 2). This result apart from being the clear confirmation of the hybrid origin of the species under study also points to the conservation of heterozygosity for these chromosomal fragments in many generations (age of the unisexual form) of the parthenogenetic *D. unisexualis*. The maternal status of *D. raddei nairensis* for the parthenogenetic *D. unisexualis* has previously been confirmed by mitochondrial DNA analysis^14,15,20^. *D. valentini* was proposed as the paternal species in several studies^10,14,20^, although this should be further confirmed by comparative cytogenetic analysis such as for example multicolor FISH.

Additionally, it seems reasonable to assume that the pericentromeric chromatin regions found on 19 chromosomes, annealed mainly with the red-labeled *D. raddei nairensis* gDNA, may represent *D. raddei nairensis*-specific pericentromeric repetitive DNAs. The clearly visible separation of parental genomes, as highlighted in Fig. 2, is a well-known feature in vertebrates^59^.

In our previous study, we had detected double centromere signals in several meiotic chromosomes in the *D. raddei* male karyotype^40^. Additional centromeres in the chromosomes may be associated with the formation of the so-called centromere environment, usually consisting of satellite tandemly repeated DNA elements^60^. Species-specific centromere organization in the *D. raddei* karyotype may be connected with the red pericentromeric signals detected on 19 chromosomes of *D. unisexualis* in our current CGH experiments. On the other hand, the mechanism of double centromere formation in *D. raddei* may involve epigenetic chromatin modification as far as a high degree of variability was shown for the formation of additional immunostaining centromere signals on the certain chromosomes^40^.

An important conclusion that can be made based on our CGH data is that no considerable exchange of DNA loci within the identified DNA fragments (red signal colors) between two homeologous (parental) sets of chromosomes seems to have occurred in the *D. unisexualis* hybrid karyotype. It is known, that centromere has crossing-over interference activity in meiosis, reducing the probability of crossing-over in the regions close to centromeres^61,62^. This is consistent with our results of CGH in the pericentromeric regions on half of the chromosomes in the *D. unisexualis* somatic karyotype (Fig. 2).

Therefore, at present time we may detect only the «residual traces» of the differences between the two chromosome sets belonging to the parental species, which are preserved explicitly in the pericentromeric chromosome regions that were not involved in the process of possible (see below) crossing-over between the homeologous chromosomes.

### Meiotic prophase I stages in maternal bisexual species *D. raddei nairensis* and parthenogenetic species *D. unisexualis*

It is known that meiosis constitutes a serious obstacle to the production of fertile progeny by interspecific hybrids. The studies of meiotic prophase I in mammalian interspecies hybrids have demonstrated that when chromosome synapsis is disturbed, cells undergo selection at the pachytene stage^63^ and are removed by apoptosis. Synapsis between the heteromorphic sex chromosomes is of especial value with the defects in synapsis or at least abnormalities in common sex body formation between heteromorphic sex chromosomes leading to aneuploidy in the germ cells^63,64^.

Studies of oogenesis in reptiles reported so far were mainly performed using the histological sections of ovarian tissue^34,35,65,66^. In some of them, squashed preparations were analyzed, or ovarian tissue was examined directly using confocal microscopy^24,25,67,68^. Such studies allow to assess the main parameters of oogenesis, however, it appears practically impossible to understand the peculiarities of the individual chromosomes behavior in meiotic prophase I. Using the preparations of this type, it is challenging to obtain any detailed information about the synapsis of homeologous chromosomes and the formation of chiasmata.

Our study of oogenesis in reptiles is the first to be performed using immunostaining on the preparations of spread primary oocyte nuclei.We traced the key stages of prophase I in *D. raddei nairensis* female (Fig. 3) and in *D. unisexualis* (Figs. 4 and 5). To determine every stage we used a combination of stage-specific morphological criteria similar to our previous studies in *Darevskia*^39,40^.

### Synapsis of homeologous chromosomes and assembly of synaptonemal complexes in the *D. unisexualis* hybrid karyotype

When we examined the micrographs of the successive stages of meiotic prophase I in *D. unisexualis* (Fig. 4a–i), we could observe no obvious disturbance of the synaptic dynamics in the 18 autosomal pairs, while the pair of sex chromosomes (Z and w) did not synapse. We also confirmed the fact that the synapsis of chromosomes at the zygotene stage in *D. unisexualis* is associated with DSB repair (Fig. 5d), similar to the results obtained in our previous study performed in the parental species males^39^.

Thus, synapsing chromosomes of *D. unisexualis*, which are homeologous, demonstrate not only the limited (partial) peritelomeric synapsis, described previously in the diploid parthenogenetic species of the genus *Aspidoscelis* (*Cnemidophorus*)^25^, but also the full-length synapsis. In contrast, in the diploid parthenogenetic reptile *Aspidoscelis neomexicana*, the synapsis of homeologous chromosomes during the early meiotic prophase I is possible only in small peritelomeric chromosome regions (possibly due to the large degree of karyotype divergence in the parental species). As a result, the development of primary oocytes after the late zygotene stage is blocked in *A. neomexicana*. Both divisions of meiosis in *A. neomexicana* may be completed only by those cells in which duplication of chromosomes and their synapsis (non-homeologous) occur at the early stages of meiosis^25^.

It has been demonstrated that the SC-karyotypes of the two parental species for *D. unisexualis* (*D. valentini* and *D. raddei nairensis*) have similar characteristics, consisting of 19 acrocentric SC-bivalents. However, certain differences in the dynamics of chromosome synapsis and the average number of crossing-over sites have been observed in these two parental species^39^ including the presence of additional centromeric signals on some SC-bivalents of *D. raddei*^40^ as well (Fig. 3c).

It may be assumed that after the primary act of interspecific hybridization and the production of hybrid individuals, germ cells enter meiotic prophase I and here possible problems associated with the synapsis of homeologous chromosomes, as well as problems in chiasmata formation may emerge.

However, we have shown earlier that during meiosis in the allotriploid hybrid *D. unisexualis x D. valentini* males, extended asynaptic zones in the structure of SC trivalents formed by homeologous chromosomes as well as incomplete double-strand breaks (DSBs) repair did not interfere with cells completing both meiotic divisions^39^. In this regard, it would seem reasonable to assume that in a hybrid individual formed via the cross between *D. valentini* male and *D. raddei nairensis* female, which once gave rise to parthenogenetic population, the germ cells entering meiosis were able to overcome the above-mentioned problems of synapsis and incomplete DSB repair problems. This is confirmed by the large proportion of oocytes we have observed at the diplotene stage, in contrast to the case with the parthenogenetic *A. neomexicana*, where only the cells with chromosomal duplication and «pseudobivalent» formation can reach the diplotene stage^25^.

The completion of prophase I without premeiotic chromosome duplication is facilitated by the absence of strict meiotic checkpoints, previously described in rock lizards^39^ and birds^69^. In rock lizards, this is confirmed not only by the identification of cells at all meiotic prophase I stages, but also by the formation of a large number of mature spermatozoa in triploid male hybrids (3n = 57, 54 A + ZZw), albeit with morphological abnormalities^39^.

Therefore, we may speculate that the above-mentioned characteristics of meiosis in rock lizard, the reduction or complete absence of checkpoints, and the similarity of parental karyotypes, represent the basis for the formation of the *D. unisexualis* hybrid karyotype.

During subsequent generations, the processes associated with overcoming the so-called “genomic shock”^70^, also called “genomic stress” in many of publications^70–72^, may have followed. This term was suggested to describe the mechanisms that «forced the genome to restructure itself in order to overcome a threat to its survival»^70^. Most works are devoted to describing this phenomenon in allopolyploid plants^73,74^ or animals^72,75–78^. However, several works on genomic shock (or stress) in diploids of hybrid origin have also been performed^71,72,79,80^. Genomic shock refers to events that constitute the early stages of interaction between two different genomes and are associated with the high heterozygosity of the newly formed hybrid karyotype^76^. Several studies have shown that the genomic shock condition caused by the formation of a new hybrid karyotype may be accompanied by multiple genetic and epigenetic changes: elimination of chromatin^74^, changes in gene expression^81^, epigenetic destabilization of genomic repeats^75,82^, and the expansion of transposons^83^. The next stage following hybridization, the hybrid genome stabilization, occurs in several subsequent generations^83,84^.

### Sex chromosomes of maternal bisexual species *D. raddei nairensis* and parthenogenetic species *D. unisexualis*

We identified Z and w sex chromosomes in *D. raddei nairensis* and *D. unisexualis* based on the following observations:

1. Identification of two asynapted axial elements with different lengths, following the data on the reduced type of w chromosome inherited from the maternal *D. raddei* species^49,50^.
2. The axial elements of these two chromosomes are thickened (Figs. 3b,c and 4g–i), which makes them distinguishable from SC-bivalents by immunostaining with the anti-SYCP3 protein antibodies and which is characteristic of the sex chromosomes of many animal species^85–88^.
3. Immunostaining with antibodies against centromere proteins (ACA) revealed that the ACA-foci on this asynapted chromosome pair look smaller than those formed by synapsed autosomal SC-bivalents, where closely located centromeres give doubled or brighter signal^85^ (Figs. 3a,c, 4h,i and 5b). The total number of ACA foci per nucleus in both species is 20, which corresponds to the signals on 18 autosomal chromosomes and one small signal on each of Z and w chromosomes, respectively.
4. FISH with *D. raddei nairensis* gDNA at the diplotene stage in *D. unisexualis* revealed a strong signal on the W chromosome pericentromeric region, but not on Z chromosome (Fig. 6c). This result corresponds with the fact that the heterochromatin-enriched w chromosome was inherited from the maternal species *D. raddei nairensis*^89,90^.

In *D. raddei nairensis* two variants of sex chromosomes location on the synaptonemal complexes spread preparation were observed: sex chromosomes located closely (Fig. 3b), and asynaptic w and Z univalents (Fig. 3c). In *D. unisexualis* we found the absence of synapsis between the Z and w chromosomes at the pachytene and diplotene stages (Fig. 4g–i).

It is known that synapsis and chiasma formation between heteromorphic sex chromosomes is important for balanced gamete production. Disruption of synapsis and the absence of chiasma between sex chromosomes can lead to the formation of aneuploid germ cells. The question that arises is: ‘How is asynapsis of sex chromosomes compensated for in the parthenogenetic species analyzed in this study?’

In most organisms studied so far, a chiasma is observed between the sex Z and W chromosomes, which often have a small zone of homology (pseudoautosomal region, PAR). The formation of a single recombination nodule in the ZW bivalent in chicken and quail has been described in the PAR region very close to the telomeric end^91^. Recent studies of oogenesis in chicken have revealed that asynapsis of the Z and W chromosomes and incomplete repair of DSBs normally occurs in 20% of primary oocytes. Oocytes carrying these asynapsed bivalents are, however, able to pass to the diplotene stage^69^. The authors, hence, suggested the lower efficiency or complete absence of a checkpoint (possibly its secondary loss during evolution) in meiotic prophase I in bird oogenesis. The absence of strict checkpoints in meiotic prophase I was also suggested by us in triploid hybrids (3n = 57, 54 A + ZZw) of *D. unisexualis x D. valentini* rock lizards^39^.

It is possible that synaptic Z and w chromosomes were not detected because of the differences in the PARs acquired during the evolution of the rock lizard parental species. As it is known that the w chromosome of the maternal species *D. raddei* has been significantly shortened during evolution, and it is referred to as the progressive w chromosome type^90^, in contrast to the ancestral type of W chromosome found in other bisexual species of rock lizards studied to date (*D. valentini*, *D. portschinskii*).

### The mechanism of ploidy restoration during oogenesis in diploid parthenogenetic *D. unisexualis* species

Based on our analysis of chromosome synapsis during meiotic prophase I (Fig. 4), we concluded that unlike previously demonstrated by Newton *et al*.^25^ for the diploid parthenogenetic species *A. neomexicana*, premeiotic chromosome duplication in the rock lizard *D. unisexualis* does not occur. This is consistent with the data of Darevsky & Kulikova (1962; 1964)^34,35^, who showed that the most closely related parthenogenetic rock lizard species *D. armeniaca* has normal meiosis (similar to the females of the bisexual species *D. raddei nairensis*). In such parthenogenetic females, the first polar body and the metaphase plate of the second division are formed during the first reduction division. However, the authors were unable to detect the second polar body^34,35^. Based on these observations, it was suggested that diploidy during oogenesis in *D. armeniaca* is restored during the subsequent stages of meiosis. Terminal fusion and gamete duplication mechanisms could not support high levels of heterozygosity between generations^92^. As the most probable mechanism for diploidy restoration, in the absence of fertilization is the fusion of haploid pronucleus with one of the haploid descendants of the first polar body was proposed^36^, the so-called “central fusion”.

“Central fusion” implies the reunification of Z and w sex chromosomes, which previously segregated in the meiotic anaphase I, in the same nucleus^93^. We cannot also exclude the variant where problems with Z and w segregation may arise during anaphase I because of sex chromosomes asynapsis. For example, both sex chromosomes may remain in the pronucleus resulting in polar body lacking sex chromosomes. But in the case of the “central fusion”, it is possible to restore the full set of chromosomes, even with the temporary cell aneuploidy at the intermediate stages of this process.

Moreover, diploidy restoration through the “central fusion” explains the preservation of a heteromorphic autosomal chromosome pair in one of the parthenogenetic species – *D. rostombekowi*^36^. Our results are overall similar to what had been found for parthenogenetic species *D. armeniaca*^33–35^.

**Figure 7:**
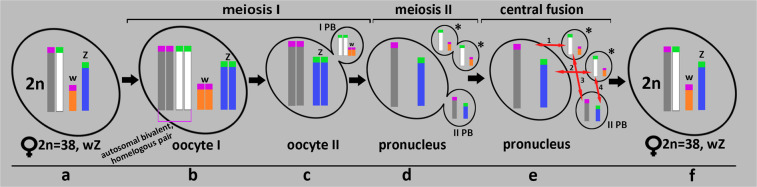
Scheme of possible automictic variants during the oogenesis of the parthenogenetic species *D. unisexualis* (according to Darevsky & Kulikova (1962), Darevsky *et al*., (1973), and our own results presented here). (**a**)– diploid karyotype of *D. unisexualis* (2n = 38). Grey and white represent members of a homelogous pair. Green and violet squares represent centromere regions inherited from *D. valentini* and *D. raddei nairensis* respectively. W and Z - sex chromosomes. (**b**)– Oocyte I contains 18 autosomal bivalents (pairs of homeologs) and two asynapsed sex univalents (w and Z). (**c**)– Result of the first meiotic division of oocyte I - 18 autosomes and one of the sex chromosomes goes into oocyte II (Z on this scheme). In the first polar body (I PB) there are 18 autosomes and another sex chromosome (here - w chromosome). At this stage, each chromosome consists of two chromatids. (**d**)– Result of the second meiotic division, 18 autosomal univalents and Z univalent enter the pronucleus. Additionally, in the second polar (II PB) body there are 18 autosomal univalents and the sex w chromosome. All chromosomes consist of one chromatid. (**e**)– Possible variants of fusion between pronucleus and one of the haploid first polar body descendants (asterisk) are indicated by the numbers 1–4. (**f**)– Result of the «central fusion»: restoration of dipolidy 2n = 38 of the formed egg, maintanance of homeologous chromosomes pairs, the reunification of Zw chromosomes pair leading to all female progeny.

We present below the schematic representation of the possible automictic process variants which may take place during oogenesis in the parthenogenetic species *D. unisexualis* considering our new data: specifics of the pericentromeric regions of hom**e**ologous chromosomes and asynaptic Z and w sex chromosomes of *D. unisexualis* (Fig. 7).

### The possibility of meiotic recombination in parthenogenetic species *D*. *unisexualis*

Evolutionary processes in automictic populations are studied in the context of crossing-over and its effects on heterozygosity levels in offsprings^92^. As described above, the diploid parthenogenetic reptile *Aspidoscelis neomexicana* overcomes meiosis by chromosome duplication and synapsis of “pseudo-bivalents”. This mechanism excludes recombination between homeologs. Surprisingly, the level of fertility in such animals is comparable with that in their bisexual relatives^25^. This is believed to be necessary to maintain the heterozygous state of the organism through the generations^12,80^. Comparative studies of allozyme data in bisexual and parthenogenetic species of rock lizards revealed high heterozygosity levels in the latter, which may indicate the suppression of meiotic recombination^7^.

We have shown that in the *D. unisexualis* parthenogenetic rock lizard species, oocytes enter the prophase I of meiosis in the normal way (without ploidy duplication). Moreover, no obvious disturbance of the axial elements assembly in the autosomes were revealed (Fig. 4). At the pachytene stage, we did not detect any asynaptic zones (Fig. 4f).

The most intriguing question obviously is the question whether chiasmata form between the homeologous chromosomes of *D. unisexualis*. If it is so, what is the pattern of the distribution of the crossing-over sites? Earlier, we have demonstrated that the studied parental species populations differ in the average number of crossing-over sites per spermatocyte nucleus^39^. It was also shown that in back-cross triploid hybrid rock lizard males, 1–2 foci of MLH1 protein, the crossing-over marker, could be found in the open trivalent homeologous chromosomes^39^.

If we assume that in every generation of the parthenogenetic form, normal crossing-over occurs between pairs of homeologous chromosomes, then during the lifetime of this form, heterozygosity could be maintained longer at those loci which are nearer to the centromere^37,94^. Perhaps it was this phenomenon (residual traces of heterozygosity) that we were able to identify in the somatic karyotype of *D. unisexualis* using CGH (Fig. 2). Otherwise, in the parthenogenetic species with preserved synapsis of homeologous chromosomes, a new mechanism of meiotic recombination suppression should exist and its study being of great interest.

Our further studies will focus on the immunostaining patterns of DSBs repair markers at the late prophase I and crossing-over markers in parthenogenetic rock lizard species. These studies will help us understand the relationships between unisexual reproduction and the levels of heterozygosity in parthenogenetic rock lizards.

The results of this study are extremely important for understanding the mechanisms of heterozygosity maintenance in hybrid karyotypes of diploid parthenogenetic species, and in general, to explain the genetic basis of the widespread distribution of unisexual animal populations.

## Conclusions

1. The distribution of species-specific pericentromeric chromatin regions of the two parental species has been demonstrated for the first time in a hybrid somatic karyotype of the parthenogenetic rock lizards species using CGH.
2. The immunocytochemical study of chromosomal synapsis in meiotic prophase I was performed in the female of the maternal species *D. raddei nairensis* and parthenogenetic species *D. unisexualis* for the first time. We have confirmed the true synapsis of chromosomes during meiotic prophase I in *D. unisexualis* using antibodies to the SYCP1 protein (synaptonemal complex central element protein). The number of *D. unisexualis* chromosomes of entering synapsis in the zygotene and detected in diplotene stage corresponds to that in the parental species.
3. Asynapsis of the sex chromosomes (Z and w) has been found at the late stages of the prophase I of meiosis in the parthenogenetic species *D. unisexualis*.
4. Possible mechanisms of hybrid karyotype formation are discussed, and possible ploidy restoration pathways in automixis are proposed. Our scheme of possible automictic process describes reunification of segregated autosomal homeologous chromosomes and Z, w chromosomes in one resulting nucleus with restored diploidy according to the «central fusion» mechanism.

## Materials and Methods

One adult *D. unisexualis* individual was captured in Hrazdan population by M.S. Arakelyan and examined in August 2018. One adult *D. raddei nairensis* female was captured in Amberd population in June 2019 and examined in December 2019. The specimens were deposited in the research collection of the Yerevan State University (Hrazdan population, YSU_RC_129, specimen VS0075; Amberd population, YSU_RC_135, specimen VS0215). The manipulations with the animals followed the international rules of the Manual on Humane Use of Animals in Biomedical Research. All experimental protocols were approved by the Ethics Committee for Animal Research of the Vavilov Institute of General Genetics (protocol No. 3, November 10, 2016) in accordance with the Regulations for Laboratory Practice. Mitotic chromosomes were prepared from the bone marrow and spleen according to Ford and Hamerton, 1956^95^ with modifications and fixed in the ice-cold acetic acid-methanol solution (1:3).

Total genomic DNA (gDNA) was extracted from the liver tissue of the *D. valentini* female (Sepasar population, ZMMU R-11920/12) and *D. raddei nairensis* female samples (Arzni population, ZMMU R-16042) from the collection of the Zoological Museum of the Lomonosov Moscow State University (ZMMU) using the DNA-extran-2 kit (EX-511, Syntol, Russia). The gDNA of *D. raddei nairensis* was directly labeled with Atto550 (red) using the Nick-translation labeling kit (Jena Bioscience), while the gDNA of *D. valentini* was labeled with Atto488 (green) using the Nick-translation labeling kit (Jena Bioscience) as well. Both gDNA were hybridized against the chromosomal background of *D. unisexualis* following the CGH protocol described in Symonová *et al*.^96^. It should be emphasized that unlabelled Cot-1 DNA (DNA fraction enriched with DNA repeats) was intentionally not used in the experiments since the alternative CGH variant performed with the presence of Cot-1 DNA in the hybridization mixture appeared to be unable to reveal significant differences in the hybridization patterns.

Germinal beds were isolated and disaggregated in the Eagle medium (C-160, Paneco, Russia). Squashed primary oocytes were prepared and fixed using the technique developed by Page *et al*.^97^, the residual cell suspension was used for the spread oocytes nuclei preparations according to Navarro *et al*.^98^. Poly-l-lysine-coated slides were used in all immunofluorescence studies. The slides were washed with phosphate-buffered saline (PBS) and incubated overnight at 4 °C with primary antibodies diluted in the antibody dilution buffer (ADB: 3% bovine serum albumin, and 0.05% Triton X-100 in PBS). Axial elements of meiotic chromosomes were detected by rabbit polyclonal antibodies against the SYCP3 protein (1:250; Abcam ab15093, Cambridge, UK), central elements of assembled synaptonemal complexes were detected by the rabbit polyclonal antibodies against the SYCP1 protein (1:200; Novus Biologicals NB300–229SS, USA). Synaptonemal complexes assembly was traced by sequential immunostaining with the anti-SYCP1 protein antibodies (SC central element protein) and anti-SYCP3 protein antibodies (SC axial elements protein) on the same nuclei. Manifestation of SYCP1 and SYCP3 proteins on meiotic chromosomes is the reliable test for synaptonemal complexes assembly. SYCP1 protein detected in zygotene-pachytene was the confirmation of assembled synaptonemal complexes – true synapsis of homeologous chromosomes^51,99^. Centromeres were detected using the anti-kinetochore proteins antibodies ACA (1:500; Antibodies Incorporated 15–234, Davis, CA, USA). After washing in PBS, the secondary antibodies diluted in ADB were used, namely, goat anti-rabbit immunoglobulin G (IgG), Alexa Fluor 488 (1:500; Abcam, Cambridge, UK), goat anti-rabbit Alexa Fluor 488 (1:500; Invitrogen, Carlsbad, CA, USA), and goat anti-human Alexa Fluor 546 (1:500; Invitrogen, Carlsbad, CA, USA). Incubation with secondary antibodies was performed in a humid chamber at 37 °C for 2 h. We used the combination of immunostaining and FISH techniques (immuno-FISH) in the following order: immunostaining with antibodies; DNA FISH on the same slide; merging the images^40,100^.

Slides were examined using the AxioImager D1 microscope (Carl Zeiss, Germany) equipped with the Axiocam HRm CCD camera (Carl Zeiss, Germany) and Carl Zeiss filter sets (FS01, FS38HE, and FS43HE), and processed with the aid of the image-processing AxioVision Release 4.6.3. software (Carl Zeiss, Germany). All preparations were mounted in Vectashield antifade mounting medium with DAPI (Vector Laboratories H-1200, Burlingame, CA, USA).

Meiotic prophase I stages were identified by analyzing the combination of basic morphological criteria used in the meiotic cell studies^51,101^. The rock lizards-specific features of the prophase I stages have been described before^39,40^. Early meiotic prophase I stages criteria for leptotene: multiple fragments of unpaired axial elements and for zygotene: long partially synapsed axial elements, «bouquet» formation (telomere clustering), and no signs of desynapsis in telomere regions. Mid-prophase I stage (pachytene) criteria: complete chromosome synapsis, and non-fragmented lateral elements of synaptonemal complexes. Diplotene criteria: signs of SCs disassembly (desynapsis of the lateral elements start in peritelomeric or interstitial regions, elongation, and fragmentation).

## Supplementary information

Supplementary Information.

## Data Availability

The following information was supplied regarding data availability: 10.5281/zenodo.3611475.
