# Supplementary material for: Cytogenetic mechanisms of unisexuality in rock lizards
Source: Sci Rep. 2020 May 26;10:8697. doi: 10.1038/s41598-020-65686-7 (PMC7250862; doi:10.1038/s41598-020-65686-7)
Supplement: Supplementary file 1 — Supplementary Information. [file 41598_2020_65686_MOESM1_ESM.doc]

**Supplementary Information**

**Cytogenetic mechanisms of unisexuality in rock lizards**

**Victor Spangenberg^1^, Marine Arakelyan^2^, Marcelo de Bello Cioffi^3^, Thomas Liehr^4^, Ahmed Al-Rikabi^4^, Elena Martynova^5^, Felix Danielyan^2^, Ilona Stepanyan^6^, Eduard Galoyan^7^, Oxana Kolomiets^1^.**

^1^ Vavilov Institute of General Genetics RAS, Moscow, Russia;

^2^ Department of Zoology, Yerevan State University, Yerevan, Armenia;

^3^ Laboratório de Citogenética de Peixes, Departamento de Genética e Evolução, Universidade Federal de São Carlos, São Carlos, SP, Brazil;

^4^ Institute of Human Genetics, Jena University Hospital, Friedrich Schiller University;

^5^ Skolkovo Institute of Science and Technology, Moscow, Russia;

^6^ Scientific Center of Zoology and Hydroecology, Yerevan, Armenia;

^7^ Severtsov Institute of Ecology and Evolution RAS, Moscow, Russia.

The following information was supplied regarding data availability:

Victor Spangenberg. (2020, January 17).

Raw data. Zenodo. <http://doi.org/10.5281/zenodo.3611475>


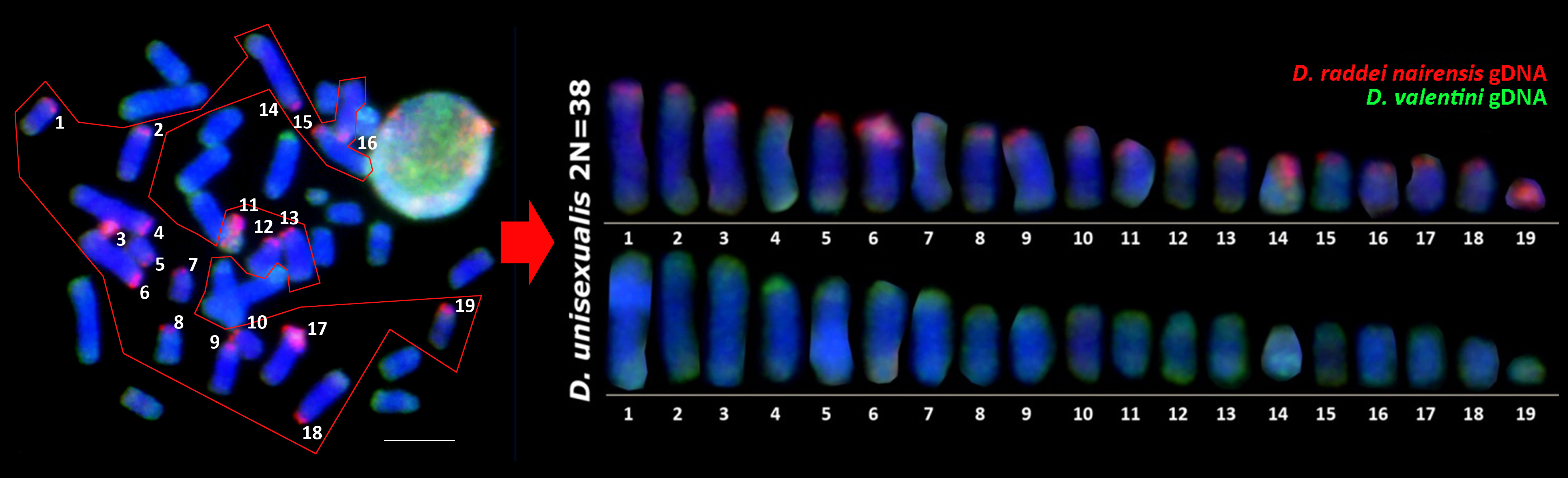


**Figure 2 variant 2**. Comparative genomic hybridization (CGH) on the metaphase plate of the parthenogenetic species *D. unisexualis* (2N = 38) with the DNA-FISH probes for the genomic DNA of the two parental species *D. raddei nairensis* (red) and *D. valentini* (green).
